# Supplementary material for: Comparative multiomics analysis of cell physiological state after culture in a basket bioreactor
Source: Sci Rep. 2022 Nov 23;12:20161. doi: 10.1038/s41598-022-24687-4 (PMC9686226; doi:10.1038/s41598-022-24687-4)
Supplement: Supplementary file 1 — Supplementary Information 1. [file 41598_2022_24687_MOESM1_ESM.zip › raw data/Transcriptomic raw data/6.Enrichment/1.GO/TE1vsTC1/ALL/TE1vsTC1.all.GOmf_DAG.pdf]

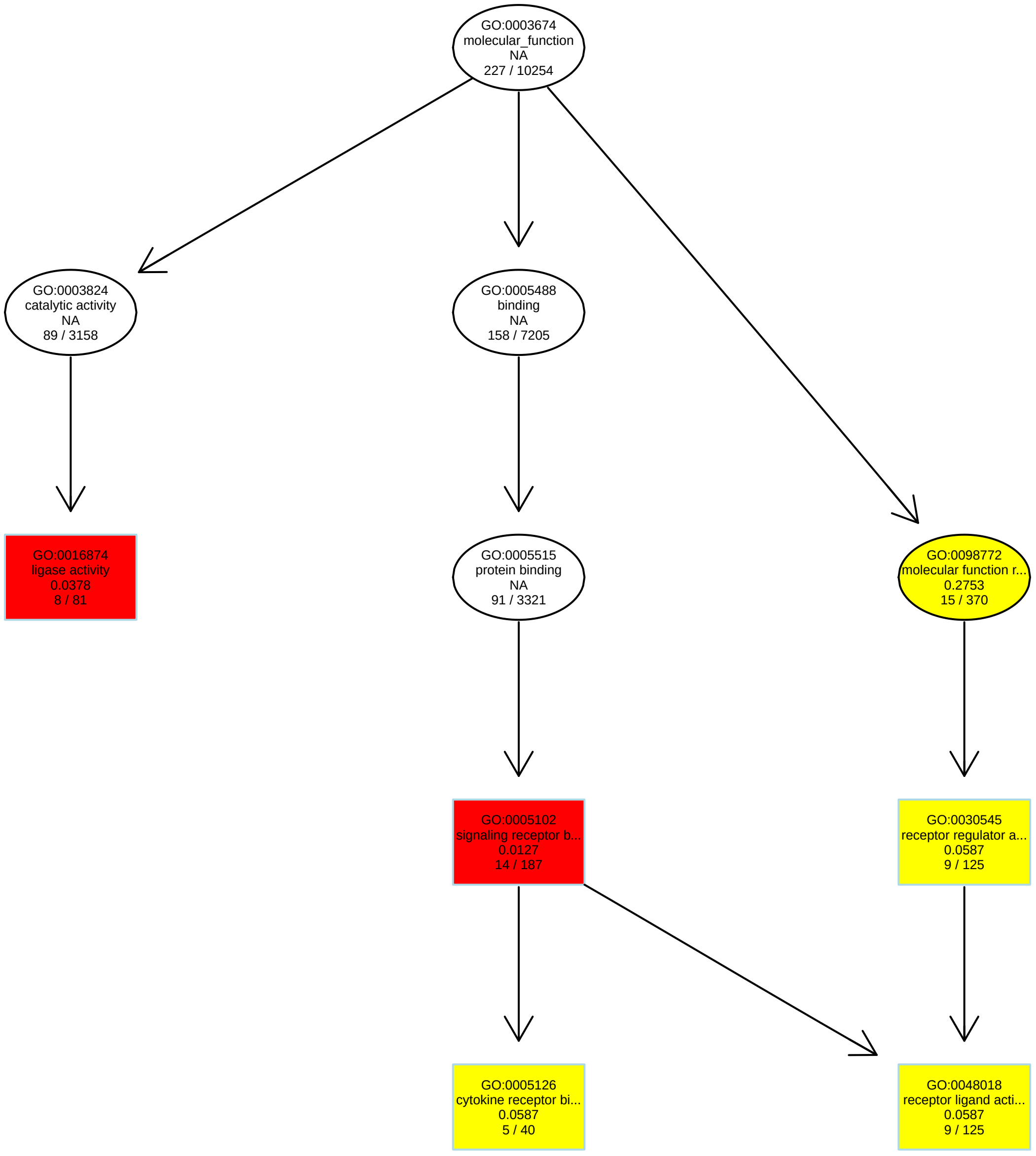
GO:0003674
molecular_function
NA
227 / 10254
GO:0003824
catalytic activity
NA
89 / 3158
GO:0005488
binding
NA
158 / 7205
GO:0016874
ligase activity
0.0378
8 / 81
GO:0005515
protein binding
NA
91 / 3321
GO:0098772
molecular function r...
0.2753
15 / 370
GO:0005102
signaling receptor b...
0.0127
14 / 187
GO:0030545
receptor regulator a...
0.0587
9 / 125
GO:0005126
cytokine receptor bi...
0.0587
5 / 40
GO:0048018
receptor ligand acti...
0.0587
9 / 125
